# Quantitative Expression of C-Type Lectin Receptors in Humans and Mice

**DOI:** 10.3390/ijms130810113

**Published:** 2012-08-14

**Authors:** Maciej Lech, Heni Eka Susanti, Christoph Römmele, Regina Gröbmayr, Roman Günthner, Hans-Joachim Anders

**Affiliations:** Medical Clinic and Policlinic IV, Nephrological Center, University of Munich, 80336 Munich, Germany

**Keywords:** infection, pattern recognition receptors, innate immunity, inflammation, macrophages, dendritic cells

## Abstract

C-type lectin receptors and their adaptor molecules are involved in the recognition of glycosylated self-antigens and pathogens. However, little is known about the species- and organ-specific expression profiles of these molecules. We therefore determined the mRNA expression levels of Dectin-1, MR1, MR2, DC-SIGN, Syk, Card-9, Bcl-10, Malt-1, Src, Dec-205, Galectin-1, Tim-3, Trem-1, and DAP-12 in 11 solid organs of human and mice. Mouse organs revealed lower mRNA levels of most molecules compared to spleen. However, Dec-205 and Galectin-1 in thymus, Src in brain, MR2, Card-9, Bcl-10, Src, and Dec-205 in small intestine, MR2, Bcl-10, Src, Galectin-1 in kidney, and Src and Galectin-1 in muscle were at least 2-fold higher expressed compared to spleen. Human lung, liver and heart expressed higher mRNA levels of most genes compared to spleen. Dectin-1, MR1, Syk and Trem-1 mRNA were strongly up-regulated upon ischemia-reperfusion injury in murine kidney. Tim3, DAP-12, Card-9, DC-SIGN and MR2 were further up-regulated during renal fibrosis. Murine kidney showed higher DAP-12, Syk, Card-9 and Dectin-1 mRNA expression during the progression of lupus nephritis. Thus, the organ-, and species-specific expression of C-type lectin receptors is different between mice and humans which must be considered in the interpretation of related studies.

## 1. Introduction

Multicellular organisms recognize potentially harmful dangers and rapidly trigger appropriate defense mechanisms that prevent or minimize tissue damage [1]. Microbial pathogen-associated molecular patterns (PAMPs) or endogenous danger-associated molecular patterns (DAMPs) are often made up of carbohydrates such as glucans, mannose or fucose [2]. C-type lectin receptors (CLR) recognize such glycoproteins and initiate inflammatory responses by activating different adaptor molecules [3]. Unlike the Toll-like receptors (TLR), CLRs are able to internalise the antigens [4]. They are part of a large family of proteins that contain an extracellular carbohydrate recognition domain and calcium-binding sites. Recent studies indicate that many CLRs, such as Dectin-1 or DC-SIGN function as pattern recognition receptors. Upon ligand binding CLRs activate signal transduction involving Syk- and Card-9-dependent activation of nuclear factor- B (NF-κB) and contribute to innate immunity and tissue inflammation. The current evidence on the functional roles of these molecules in host defense and danger control largely derives from *in vitro* studies or experiments using knock-out mice. Pattern recognition receptor genes show large homologies among species, therefore, data originating from mice often predict similar functions in humans [5]. However, important discrepancies exist. For example, humans lack functional TLR11 [6], mice lack TLR8 [7], and TLR9 is expressed in murine but not in human macrophages [8]. Similarly, there are also different functions of CLR in mice and humans. For example, murine DC-SIGN is down-regulated upon activation in dendritic cells, but unlike human DC-SIGN, incubation with IL-4 and IL-13 does not enhance mouse DC-SIGN expression [9]. Mouse DC-SIGN is not involved in T cell–DC interactions and it does not bind to pathogens known to interact with human DC-SIGN, such as *Leishmania mexicana*, cytomegalovirus, and HIV [9]. While the expression of the TLRs and cytosolic PRRs have been extensively characterized [10,11] little data are available on the species-specific expression of CLRs and their adaptor molecules. We hypothesized that CLRs and CLR-related molecules are differentially expressed in humans and mice; hence, we characterized their mRNA expression profiles in human and murine solid organs as well as in a number of disease models that involve innate immune responses.

## 2. Results and Discussion

### 2.1. C-Type Lectin Receptor mRNA Expression in Adult Human Tissues

**Figure 1:**
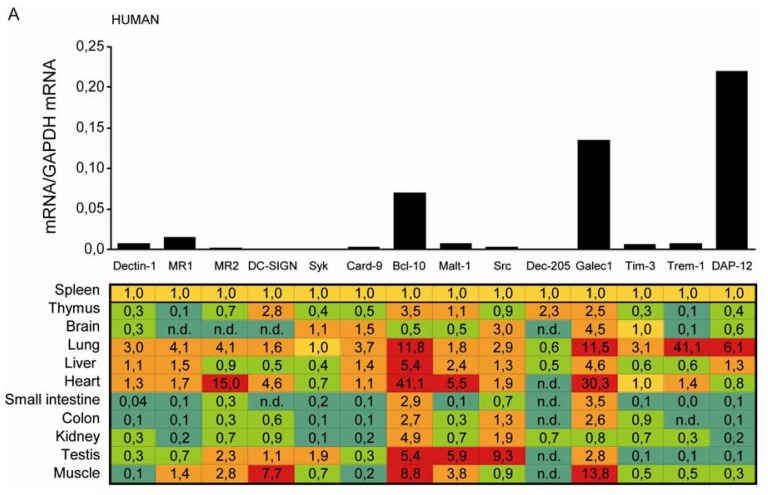

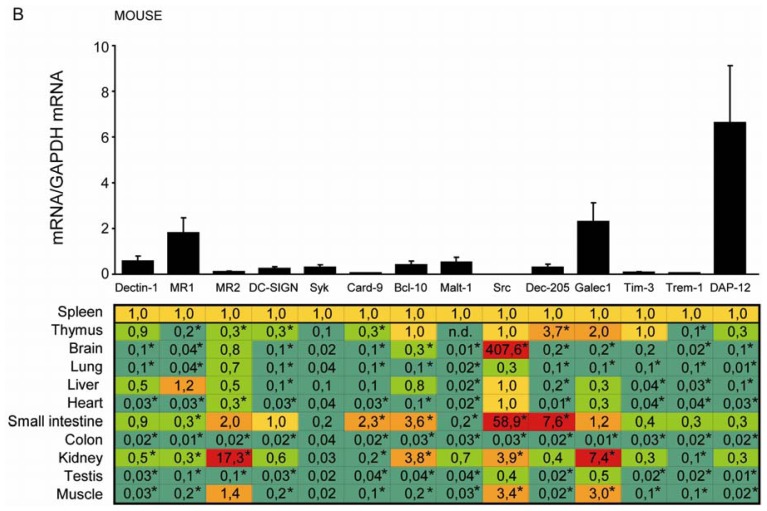
(**A**) C-type lectin receptors (CLR) mRNA expression in adult human tissues. Quantitative real-time PCR analysis was performed on pre-normalized cDNAs derived from poly(A)-selected DNase-treated RNAs purified from pools of healthy human tissues as described in methods; (**B**) CLR mRNA expression in adult mouse tissues. Real-time PCR was performed on pooled cDNAs derived from five adult six to eight week old C57BL/6 mice as described in methods. Biological replicates were quantified in triplicates and normalized to the respective GAPDH mRNA level. The results in the table are expressed relative to the respective expression level of each transcript in spleen. In the table, red shades indicate higher and green shades indicate lower mRNA levels as compared to the respective mRNA levels in spleen. The spleen mRNA levels are illustrated in the histogram on top of the table. Data in **B** represents means ± SEM. * indicated statistical significance *p* < 0.05.

We used real time RT-PCR to assess the mRNA expression levels of the following innate pattern recognition molecules in human solid organs: Dectin-1, MR1, MR2, DC-SIGN, Syk, Card-9, Bcl-10, Malt-1, Src, Dec-205, Galectin-1, Tim-3, Trem-1, and DAP-12. Most of these molecules were constitutively expressed in human spleen (Figure 1A). DC-SIGN, Syk, and Dec-205 mRNA were only marginally detectable in spleen. In human thymus the mRNA levels of all aforementioned molecules were lower than in spleen except for DC-SIGN, Bcl-10, Dec-205 and Galectin-1. The mRNA levels of some tested receptors and signaling molecules were lower or unchanged in solid organs as compared to spleen. However human brain revealed higher levels of Src and Galectin-1. Lung revealed only lower expression of Dec-205 as compared to spleen. Expression of Bcl-10, Trem-1 and Galectin-1 were more than 10-fold higher as compared to spleen. Liver revealed substantial higher expression levels of Bcl-10 and Galectin-1, similar to small intestine and colon. All molecules except for Syk, Card-9, Dec-205, Tim-3, and DAP-12 showed higher mRNA levels in heart. Only Bcl-10 and Src were higher in kidney. Testis expresses substantially higher levels of MR2, Syk, Bcl-10, Malt-1, Src, and Galectin-1. MR2, DC-SIGN, Bcl-10, Malt-1, and Galectin-1 were found to be highly expressed in muscle. Thus, human organs display an organ-specific CLRs mRNA expression pattern.

### 2.2. C-Type Lectin Receptor mRNA Expression in Adult Murine Tissues

Next we determined the mRNA expression levels of the same molecules in the same 11 murine organs from six to eight week and six month old C57BL/6 mice. All molecules were constitutively expressed in mouse spleen but the mRNA level of Src was just above detection level (Figure 1B). In all other solid organs gene expression levels of all tested molecules were lower or similar to splenic expression levels. However, Dec-205 and Galectin-1 mRNA levels were higher in mouse thymus; Src was higher in brain, small intestine, kidney and muscle. Small intestine expressed higher levels of MR2, Card-9, Bcl-10, and Dec-205. In murine kidney only MR2, Bcl-10, Src, and Galectin-1 showed substantially higher expression.

**Figure 2:**
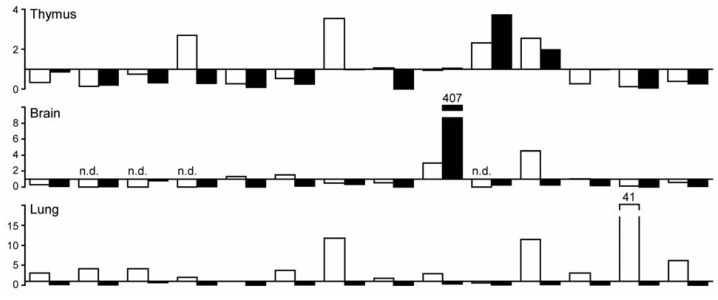

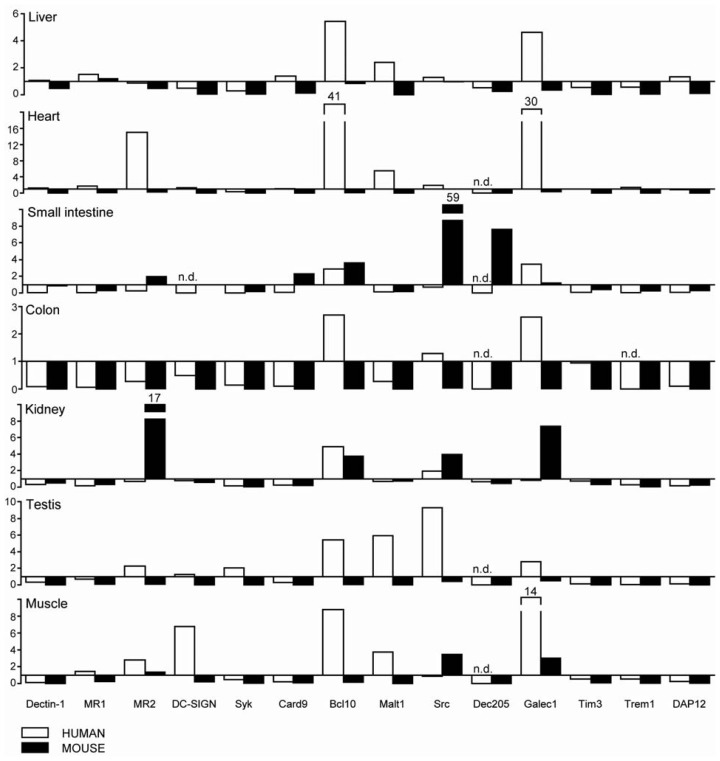
CLR mRNA expression in adult human and mouse tissues. The respective relative human (open bars) and murine (black bars) CLRs mRNA levels from figure 1A,B are illustrated. The *x*-axis marks a ratio of 1, hence, positive values indicate stronger expression in humans, negative values indicate stronger expression in mice. The *y*-axis marks the fold-change in each direction. Note that the scale of the *y*-axis is different for each organ.

Figure 2 compares the organ-specific CLRs mRNA expression in humans and mice, and illustrates direct comparison of relative mRNA expressions between the two species. White (human) and black (murine) bars indicate the x-fold induction versus respective spleen mRNA expression levels. For example, higher mRNA levels of Bcl-10 in all organs except small intestine and brain were observed in humans but not in mice. Also Galectin-1 mRNA expression was higher in humans than in mice in all the tested organs except for kidney. Human lung showed higher expression of every tested molecule in comparison to murine samples. The relative Src mRNA expression in mouse brain, small intestine, kidney and muscle was higher than in humans. Murine kidney expressed higher levels of MR2 and Galectin-1 as compared to humans. MR2, Card-9 and Dec-205 mRNA expression was higher in murine than in human small intestine. Muscle and heart also displayed almost identical relative mRNA expression patterns in humans and mice. We conclude that the mRNA expression levels of tested molecules differ in human and mouse organs.

### 2.3. C-Type Lectin Receptor mRNA Expression in Ischemia-Reperfusion Injury (IRI)

**Figure 3:**
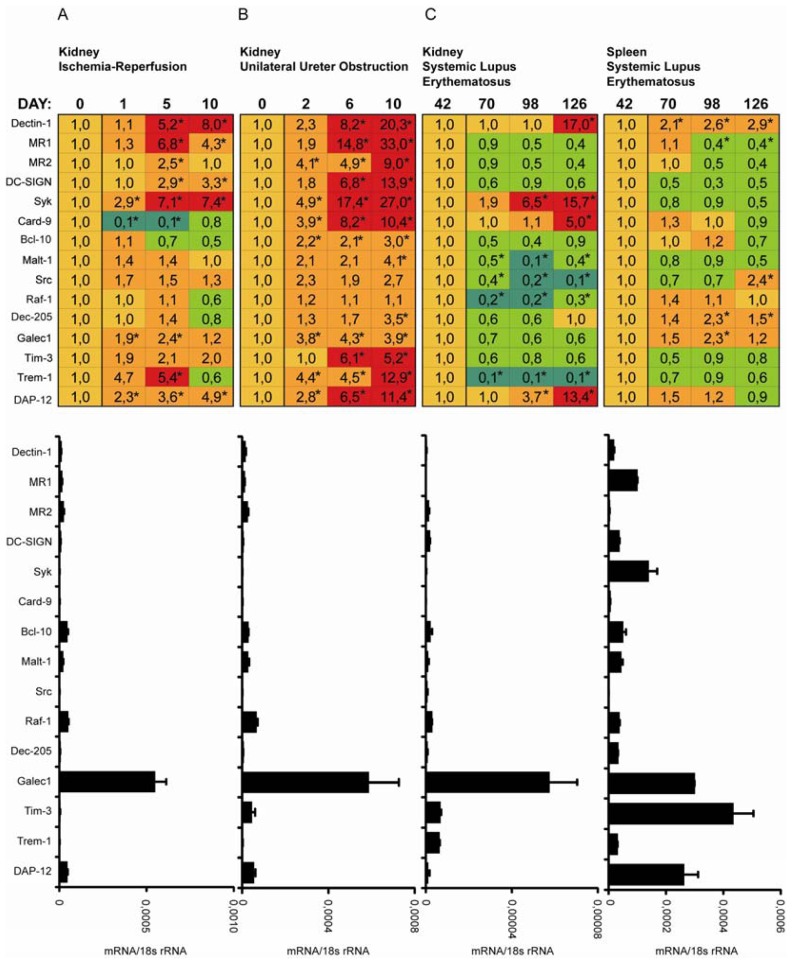
(**A**) CLRs mRNA expression in mouse kidney upon induction of IRI; (**B**) CLRs mRNA expression in mouse kidney upon induction of UUO; (**C**) CLRs mRNA expression in mouse kidney upon induction of lupus nephritis (LN). Real-time PCR was performed on cDNAs derived from murine kidney tissue from five to ten C57BL/6 mice that underwent ischemia reperfusion injury (IRI) (A), unilateral ureteral obstruction (UUO) (B) or progressive systemic autoimmunity with lupus nephritis in MRLlpr mice (C) as described in methods. Biological replicates were quantified and normalized to 18s rRNA level. The results in the table are expressed relative to the respective expression level of each transcript in sham operated kidney (A, B) or six week old MRLlpr mice (C). In the table, red shades indicate higher and green shades indicate lower mRNA levels as compared to the respective mRNA levels in spleen. Basal mRNA levels in control kidney are illustrated in the histogram below the table. Data in histograms represents means ± SEM. * In the tables indicates statistical significance *p* < 0.05.

**Figure 4:**
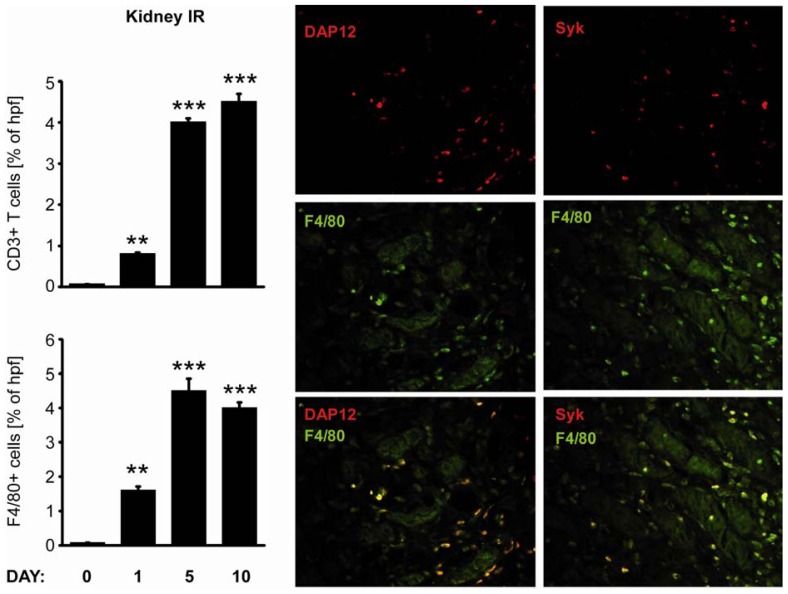
Renal leukocyte recruitment upon renal ischemia-reperfusion injury. Kidneys were obtained from all IRI mice at various time intervals as indicated. Immunostaining for macrophages and T-cells was performed on renal sections as described in material and methods. Data represents positive stained surface in percent and are shown as mean ± SEM from 8 to 12 mice (at least 25 hpf) of each group. F4/80, DAP-12 and Syk immunostainings were performed on paraffin embedded renal sections, obtained from mice 10 days after IRI, as described in material and methods. Data shows most representative results from each group. Data in histograms represents means ± SEM. ** Indicates statistical significance *p* < 0.01, *** indicates statistical significance *p* < 0.005.

Acute renal ischemia causes renal failure due to tubular cell necrosis as well as an activation of innate immunity which contributes to tissue injury and organ dysfunction [12]. We used transient renal pedicle clamping to study the expression of CLR throughout the course of ischemia-reperfusion injury (Figure 3A). Increase in mRNA expression was mostly evident for Syk, Galectin-1, Tim-3, Trem-1 and already at day one after renal artery clamping. Expression of Dectin-1, MR1, Syk and DAP-12 was higher at 10 days after injury. Card-9 and Bcl-10 were down-regulated after acute kidney injury. We conclude that Dectin-1, Syk, DAP-12 and MR1 mRNA are persistently induced upon renal ischemia-reperfusion injury which corresponds to their expression in renal mononuclear cells that persistently infiltrate the postischemic kidney, especially during the late inflammatory phase and repair phase of renal injury. It is known that oxidative stress and ischemic injury trigger the inflammatory responses. Increased renal proinflammatory chemokine and cytokine expression after ischemia reperfusion injury (IRI), supports recruitment of immune cells to sites of inflammation. In fact immunostaining revealed increased numbers of CD3+ and F4/80+ immune cells in the renal interstitial compartment 24 h after renal artery clamping. To validate the increased mRNA level, we performed co-immunostaining of Syk and DAP-12 with macrophage F4/80 marker. Co-expression of F4/80 marker with Syk or DAP-12 upon IRI was consistent with mRNA expression pattern (Figure 4).

### 2.4. C-Type Lectin Receptor mRNA Expression in Progressive Renal Fibrosis

**Figure 5:**
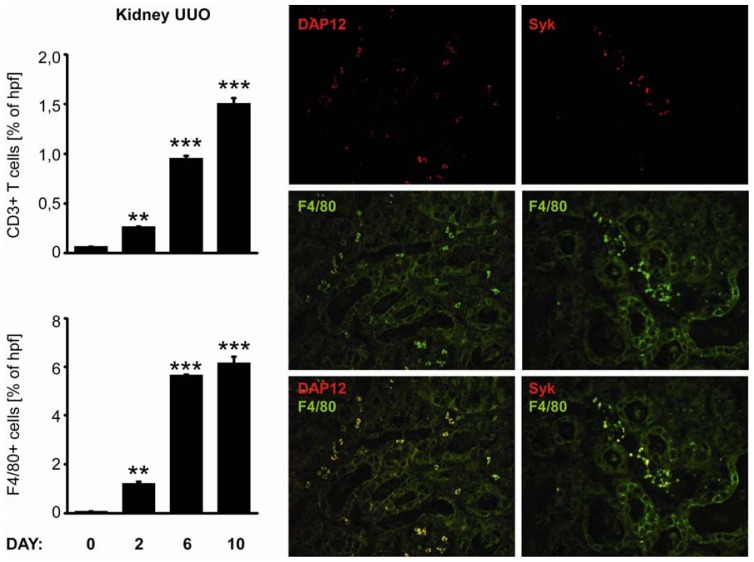
Renal leukocyte recruitment upon unilateral ureteral obstruction. Kidneys were obtained from mice after UUO at various time intervals as indicated. Immunostaining for macrophages and T-cells was performed on renal sections as described in material and methods. Data represents positive stained surface in percent and are shown as mean ± SEM from 8 to 12 mice (at least 25 hpf) of each group. F4/80, DAP-12 and Syk immunostainings were performed on paraffin embedded renal sections, obtained from mice 10 days after UUO, as described in material and methods. Data shows most representative results from each group. Data in histograms represents means ± SEM. ** Indicates statistical significance *p* < 0.01, *** indicates statistical significance *p* < 0.005.

Progressive tissue fibrosis is a common consequence of persistent sterile tissue inflammation [13]. We used unilateral ureteral ligation as a sterile model of progressive renal fibrosis. As unilateral ureteral obstruction (UUO) induced the intrarenal expression of not only proinflammatory but also profibrotic mediators, we compared renal mRNA expression level of CLRs and their adaptor molecules in obstructed kidney in relation to sham operated kidney at 2, 6 and 10 days after surgery (Figure 3B). Groups of C57BL/6 mice underwent surgery as described in material and methods. Most CLR were induced upon ureteral ligation especially Dectin-1, MR1, MR2, DC-SIGN, Syk, Card-9, Tim-3, Trem-1, and DAP-12. Immunostaining of paraffin-embedded tissue revealed increased numbers of CD3+ and F4/80+ immune cells in the renal interstitial compartment 2, 6, and 10 days after unilateral ureteral ligation. We performed co-immunostaining of Syk and DAP-12 with the macrophage marker F4/80. Co-expression of F4/80 marker with Syk or DAP-12 revealed that expression of CLRs correlates with the infiltration of F4/80 positive macrophages (Figure 5). Thus, the massive infiltration of macrophages and T cells into the interstitial compartment of the obstructed kidney is associated with a strong induction of multiple CLRs.

### 2.5. C-Type Lectin Receptor mRNA Expression in Systemic Autoimmunity and Immune Complex Glomerulonephritis

**Figure 6:**
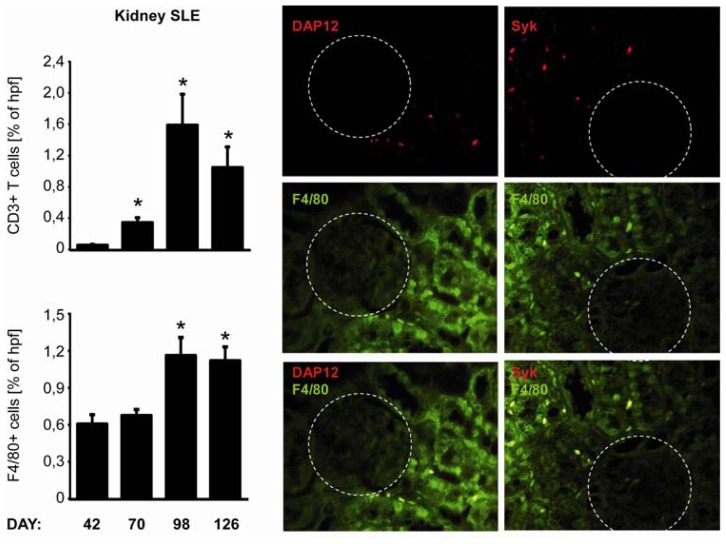
Renal leukocyte recruitment during systemic lupus of MRLpr mice. Kidneys were obtained from MRLlpr mice at various time intervals as indicated. Immunostaining for macrophages and T-cells was performed on renal sections as described in material and methods. Data represents positive stained surface in percent and are shown as mean ± SEM from 8 to 12 mice (at least 25 hpf) of each group. F4/80, DAP-12 and Syk immunostainings were performed on paraffin embedded renal sections as described in material and methods. Kidneys were obtained from 126 days old MRLlpr mice. Glomeruli are indicated by encircling them. Data shows most representative results from each group. Data in histograms represents means ± SEM. * Indicates statistical significance *p* < 0.05.

Autoimmune diseases are a consequence of immunity directed to the organism itself. We used a severe model of systemic lupus erythematosus to study expression of CLRs in kidney pathology. MRLlpr mice develop multiple auto-antibodies which results in deposition of immune complexes in kidney and renal inflammation. We analysed basal CLRs mRNA expression levels in kidney of MRLlpr mice at days 42, 70, 98 and 126 [14] (Figure 3C). The basal mRNA expression profiles of tested CLRs in young MRLlpr mice, which had not yet developed the autoimmune syndrome, were comparable to these found in C57BL/6 mice. Surprisingly, mRNA expression levels in kidneys from MRLlpr mice decreased with time in the immune complex glomerulonephritis model of autoimmune disease, even though an increased infiltration of macrophages and T-cells was observed. Only Dectin-1, Syk, Card-9 and DAP-12 mRNA expression increased in the final phase of the disease at 126 days. However, co-staining of Syk and DAP-12 with F4/80 showed only weak expression of these proteins within renal macrophages (Figure 6). Together, immune complex glomerulonephritis is associated with decreased expression of multiple CLRs, except Dectin-1, Syk, Card-9 and DAP-12, which are up-regulated in the last stages of the disease.

### 2.6. Role of C-Type Lectin Receptors in Tissue Homeostasis

Pattern recognition molecules play an important role in tissue homeostasis [15] and responses to stress signals, such as infections or tissue injury [16,17]. Some C-type lectine receptors, together with other receptors such as TLRs, nucleotide-oligomerization domain (Nod)-like receptors (NLRs) and retinoic acid-inducible gene-1 (RIG-1)-like receptors (RLRs), play a central role in innate immunity by mediating recognition of PAMPs and DAMPs. CLRs also affect antigen presentation, expression of adhesion molecules and tissue regeneration [2]. Most of the PRRs are expressed by professional antigen presenting cells such as macrophages and dendritic cells. These cells contain a set of receptors that recognise, capture, and internalise antigens [18]. Most CLRs function as antigen receptors that are particularly involved in self-antigen capture and presentation. By recruiting other signaling molecules CLRs are able not only to signal independently, but also to modulate the signaling cascade coming from other PRRs such as TLRs [18]. So far little is known about the respective expression profiles of the CLRs. Therefore, we determined their organ-specific expression and their adaptor molecules in humans and mice. We also studied the expression of single molecules in particular kidney disease models such as unilateral ureteral obstruction (UUO), ischemia reperfusion injury (IRI), and lupus nephritis (LN) both on mRNA and protein level. Previously Zarember, *et al.* described the mRNA expression of all known human TLRs, RP105, and several other molecules important in TLR function in human organs and peripheral blood leukocytes [11]. The study showed that most of the tissues express at least one TLR and spleen and PBLs express almost all TLRs. Moreover some of the TLRs appeared to be more restricted to B cells, suggesting the role of TLRs in adaptive immunity [11]. Our previous study found mRNA levels of most cytosolic pattern recognition molecules in human heart, small intestine, colon, and kidney to be lower as compared to mRNA levels in spleens [19]. This might relate to the number of immune cells in these organs. Furthermore, we observed some species-specific differences in their expression upon stimulation. Murine monocytes strongly up-regulated cytosolic pattern recognition molecules upon stimulation, whereas human monocytes down-regulated receptor expression upon stimulation [19]. Consistent with this observation and using human organ cDNA from the same tissues we found the mRNA levels of most tested CLRs and their signaling molecules in human small intestine, colon, and kidney to be lower as compared to spleen. However, lung expressed higher levels of selected molecules, except for Dec-205 as compared to spleen. High expression of Dec-205 correlates with the increased amount of lymphoid-related regulatory type of DC [20]. Expression of this receptor was only slightly detectable in human spleen, thymus, lung, liver and kidney. Mouse tissue samples showed similar expression patterns, however small intestine appeared to be an organ with a higher expression of Dec-205. Disease models did not affect the expression of Dec-205. The Syk-coupled C-type lectin receptor Dectin-1 was identified as an important receptor for the innate recognition and response to fungal pathogens [21]. Dectin-1 deficiency correlates with hypersusceptibility to mucocutaneous fungal infection in humans [21]. Consistent with its role as a pattern recognition receptor, Dectin-1 mRNA levels should be increased in tissues with an external interface. This was true for the lung; however, we did not observe significant expression in the gastrointestinal tract. After activation of Dectin-1, the Syk–dependent signaling cascade leads to an assembly of the Card-9/Bcl-10/Malt-1 complex. This process is not only activated by phosphorylation and ubiquitination, but is also influenced by Card-9 expression. Increased expression of the signaling mediator Card-9, *i.e.*, in many infections and proinflammatory conditions, might help to amplify the signal coming from surface receptors. However, activation of some receptors (for example PPAR-γ) was shown to inhibit Dectin-1–induced activation of NF-κB and MAPK signaling cascades by suppressing Card-9 [22]. Our study showed decreased or unchanged expression of all three compounds of the Syk-dependent signaling complex Card-9/Bcl-10/Malt-1 upon ischemia reperfusion injury but not upon UUO, where expression of all three mRNAs increased. Syk is a non-receptor protein tyrosine kinase that transduces signals that are involved in the control of a variety of cellular processes such as proliferation, differentiation, angiogenesis, and adhesion [23–25]. The constitutive expression levels of Syk were low in most human and mouse organs. However, the expression of Syk increased up to 27 times upon UUO as well as during renal ischemia reperfusion injury and lupus nephritis. This might be due to increased leukocyte adhesion and vascular development in injured tissue. Src signaling is also downstream of Dectin-1 activation [26]. We did not observe striking differences in expression of Src, because this molecule is probably normally maintained in an inactive state, but can be activated during mitosis [26]. High relative expression in brain and small intestine in mice results from barely detectable Src expression in spleen as the reference organ. Mouse kidney expressed high levels of MR2 which is involved in the processes of renal fibrosis [27]. MR2-deficient mice display aggravated renal fibrosis more than wild type controls which most likely results from a deregulated lysosomal collagen turnover [27]. Consistent with these data, our studies showed that MR2 expression was up-regulated upon UUO-induced renal fibrosis but not in IRI which is rather an acute tubular injury model devoid of fibrosis.

We also observed strong expression of Galectin-1 in most of the organs in humans but only in spleen, thymus, small intestine, kidney and muscle in mice. This wide expression pattern may be due to the fact that Galectin-1 can be found intracellularly, extracellularly and at the cell surface; and its varying functions depends on its location. For example, intracellular Galectin-1 is involved in the cell-cycle, RNA splicing, and transcriptional regulation; whereas extracellular and cell-surface-bound forms are involved in cell–cell interactions, immune response and apoptosis [28,29]. Surprisingly, Galectin-1 seems to be only slightly regulated during UUO, IRI or LN development. DAP-12 signaling is relevant in mature differentiated mononuclear phagocytes [30]. DAP-12 mRNA was strongly upregulated in kidneys upon UUO and in late stage lupus nephritis. Unfortunately, immunohistochemical studies that validate mRNA expression at the protein level and inform about the cellular distribution of CLRs in human tissues are mostly lacking. We observed expression levels of selected proteins upon UUO and IRI and co-localised expression within the F4/80+ macrophages as well as CD3+ cells indicating that the expressions of Syk and DAP-12 are restricted to intrarenal mononuclear phagocytes which are known to contribute in different ways to inflammation and repair of injured organs [31]. To better understand their functional contributions to the disease process interventional studies with mutant mice or suitable antagonists need to be performed applying organ-specific disease models.

## 3. Experimental Section

### 3.1. Animal Studies

Six to eight week old C57BL/6 mice were obtained from Charles River, Sulzfeld, Germany, and housed in groups of five mice with a 12 h dark/light cycle and unlimited access to food and water. Mice were sacrificed by cervical dislocation [32]. Solid organs samples were kept in RNAlater reagent and total RNA was prepared, quality checked, and reverse transcribed into cDNA (described in 3.3.). For PCR, independent pools of five samples each were used. Ischemia reperfusion injury (IRI): groups of six to eight week old C57BL/6 mice (*n* = 5–10) underwent unilateral renal pedicle clamping for 45 min as a model of ischemia-reperfusion as previously described [33]. Body temperature was maintained at 37 °C throughout the procedure by placing the mice on a heating pad. Mice were sacrificed 1, 5, and 10 days after the procedure; injured and contralateral kidneys were harvested for RNA isolation and immunohistostaining. Unilateral ureteral obstruction (UUO): groups of six to eight week old C57BL/6 mice underwent unilateral ureteral obstruction as a model of progressive renal fibrosis as previously described [34] and were sacrificed 2, 6, and 10 days after surgery. Contralateral kidneys served as intra-individual control. Autoimmune disorder: groups of female MRL or MRL/lpr mice with spontaneous lupus-like autoimmunity were housed under sterile conditions and sacrificed at age of 6, 10, 14, and 18 weeks as described [35]. All experimental procedures were performed according to the German animal care and ethics legislation and had been approved by the local government authorities.

### 3.2. Human Solid Organ cDNA

Human solid organ pre-normalized cDNAs derived from poly-(A)-selected DNase-treated RNAs were purified from pools of healthy human tissues as obtained from Clontech, Mountain View, CA. As only a single pool was available for each organ no studies on biological replicates allowing statistics could be performed. According to Clontech, all human samples were purchased and imported in accordance with all local laws and regulations.

### 3.3. Quantitative Real-Time RT-PCR

Human solid organ prenormalized cDNAs derived from poly-(A)-selected DNase-treated RNAs purified from pools of healthy human tissues were obtained from Clontech, Mountain View, CA, USA. An equal amount of cDNA from each individual preparation was used as a template in PCR with primers for each of the fours tested reference genes (α-tubulin, β-actin, GAPDH/G3PDH, phospholipase A2) (Table 1). An 18s ribosomal unit was not detectable in poly-(A)-purified RNAs. The PCR product band was determined by video imaging and computer analysis, and band intensity was determined. If necessary, the concentration of individual cDNA preparations was than adjusted so that the average band intensity for the reference genes used to normalize the panel varied no more than 20%. As only a single pool was available for each organ no studies on biological replicates allowing statistics could be performed. According to Clontech all human samples were purchased and imported in accordance with all local laws and regulations. Donors were tested to be negative for HIV, hepatitis B virus, and hepatitis C virus. Further exclusion criteria were as follows, manifest infections during the last 4 weeks, fever, symptomatic allergies, abnormal blood cell counts, increased liver enzymes, or medication of any kind except vitamins and oral contraceptives. High quality, DNA-free RNA was isolated from freshly harvested tissues. Tissues were kept in RNAlater reagent and RNA was isolated from same tissue mass (10 mg) with Pure Link RNA Mini Kit according to manufacture instructions (sample size normalization). Samples were digested with DNAse solution and additional washing steps were performed to remove traces of DNAse. Concentrations of aqueous RNA samples were measured with NanoDrop 1000 Spectrophotometer. Only samples with absorbance 260/280 between 1.95–2.05 were considered as pure RNA, the integrity of the total RNA was determined by electrophoresis on 2% (*w*/*v*) agarose gels. 1 μg of good quality RNA was preceded to cDNA (second normalization step). Thermo stable RNAse inhibitor was used during reverse transcription. Reverse transcription was performed with same reaction mix containing Superscript II reverse transcriptase (Invitrogene), dNTPs, hexanucleotides, linear acrylamid, DTT and 5× Superscript buffers using standard protocol. Same amount of RNA (1 μg) was heated to 65 °C for 5 min put on ice. RT-PCR reaction was performed for 90 min at 42 °C. Mouse cDNAs from organs and disease models were additionally normalized with α-tubulin, β-actin, GAPDH/G3PDH, phospholipase A2, β-2-microglobulin and ribosomal unit 18 s (Table 2). GAPDH was chosen for analysis of the tissues and ribosomal protein 18 s was used as a reference gene for disease models (little variation between disease models, Table 3). Above mentioned reference genes were tested in 11 (organs) or 20 (IRI, UUO or LN) samples. Geometric mean (GM), arithmetic mean (AM) minimal value, maximal value, standard deviation (SD), variance and coefficient of variance (CV) were calculated. GAPDH and 18 s were chosen as the most stably expressed in organs and disease models respectively. CLR mRNAs expression in human and mouse solid organs cDNA was quantified by real-time RT-PCR using GAPDH as housekeeper genes. Each PCR reaction (20 μL) contained 10× *Taq* Polymerase Buffer, *Taq* Polymerase, dNTPs, BSA, PCR Optimizer, SYBR green solution, MgCl_2_, gene specific primers and 0.2 μL of synthesized cDNA. SYBR Green Dye detection system (SYBR Green I 96 protocol LC480 Roche running program) was used for amplification. Quantitative real-time PCR was performed on Light Cycler 480 (Roche, Mannheim, Germany). Each amplification step included initiation phase 95 °C, annealing phase 60 °C and amplification phase 72 °C and was repeated 45 times. Gene-specific primers (300 nM, Metabion, Martinsried, Germany) were used as listed in Table 4. Controls consisting of ddH_2_O were negative for target and housekeeper genes. Primers were designed to be cDNA specific and to target possibly all known transcripts of genes of interest. *In silico* specificity screen (BLAST) was performed. The lengths of amplicons were between 80 and 130 bp. The kinetics of the PCR amplification (efficiency) was calculated for every set of primers. The efficiency-corrected quantification was performed automatically by the LightCycler 480 based on extern standard curves describing the PCR efficiencies of the target and the reference gene [ratio = E_target_^ΔCPtarget (control − sample)^/E_ref_^ΔCPref (control − sample)^]. To reduce the risk of false positive Cp the high confidence algorithm was used. All the samples that during the amplification reaction did not rise above the background fluorescence (crossing point Cp or quantification cycle Cq) of 40 cycles were described as not detected (n.d. = not detected in the figures). Crossing points between 5 and 40 cycles were considered as detectable. The melting curves profiles were analyzed for every sample to detect unspecific products and primer dimers. Products were visualized on agarose gels, extracted and analyzed for sequence.

### 3.4. Morphological Evaluation

Tissue sections from the kidneys of each mouse were fixed in 10% formalin in PBS and embedded in paraffin. Two micromolar per liter sections were stained with rat anti-F4/80 (Serotec, Oxford, UK, 1:50) and CD3 primary antibody. To count interstitial cells at least 25 high power fields (hpf, 100×) per group were analyzed. Positive areas were digitally quantified per high power field with use of Photoshop software. Data represents means ± SEM. For detection of CLRs in intrarenal macrophages immunohistochemistry was used. Rat anti-mouse F4/80 antibodies (Serotec, 1:50) were used for staining intrarenal macrophages. Rabbit polyclonal anti-Syk (Abcam, Cambridge, UK, 1:100 dilution) and rabbit polyclonal antibody raised against amino acids 1–113 of DAP-12 (Santa Cruz Biotechnology, Heidelberg, Germany, 1:100 dilution) were used. Appropriate secondary anti-rat and anti-rabbit FITC- or PE-coupled were used for visualizing the signal.

### 3.5. Statistical Analysis

Data were expressed as mean ± standard error of the mean (SEM). Comparisons between groups were performed using univariate ANOVA (posthoc Bonferroni’s correction was used for multiple comparisons). A value of *p* < 0.05 indicated statistical significance.

## 4. Conclusions

Together, we identified significant differences in the mRNA expression of CLRs and adaptor molecules in human and mouse solid organs and defined their cell type-specific expression in different inflammatory conditions. These findings can help to generate novel hypotheses on the role of described molecules in selected organ pathologies. Furthermore, this study shows that species-specific expression of single molecules needs to be considered in the interpretation of human studies and studies performed in rodents.

## Supplementary Materials



## Figures and Tables

**Table 1:** Descriptive statistics of candidate human reference genes (RG) based on their crossing point (CP) values. Abbreviations: N: number of samples; GM: the geometric mean of CP; AM: the arithmetic mean of CP; Min CP and Max CP: the extreme values of CP; SD: the standard deviation of the CP; CV: the coefficient of variance expressed as a percentage on the CP level.

	GAPDH	Actin	α-tubulin	Phospholipase A2	β-mikroglobulin
**N**	11	11	11	11	11
**GM**	20.35	17.97	18.98	20.55	16.49
**AM**	20.36	18.01	19.01	20.60	16.58
**Minimum**	19.12	17.35	17.96	18.39	13.71
**Maximum**	21.59	20.34	20.40	23.88	21.13
**SD**	0.75	1.17	1.12	1.49	1.84
**Variance**	0.56	1.36	1.26	2.21	3.38
**CV**	0.11	0.24	0.24	0.45	0.56

**Table 2:** Descriptive statistics of candidate mouse reference genes (RG) based on their crossing point (CP) values. Abbreviations: N: number of samples; GM: the geometric mean of CP; AM: the arithmetic mean of CP; Min CP and Max CP: the extreme values of CP; SD: the standard deviation of the CP; CV: the coefficient of variance expressed as a percentage on the CP level.

	GAPDH	Actin	α-tubulin	Phospholipase A2	β-mikroglobulin	18 s
**N**	11	11	11	11	11	11
**GM**	20.33	18.46	20.27	20.83	18.24	8.32
**AM**	20.34	18.49	20.29	20.88	18.37	8.40
**Minimum**	19.31	16.85	18.98	18.75	15.28	7.33
**Maximum**	21.38	20.01	21.28	23.84	21.07	11.22
**SD**	0.61	1.03	0.75	1.58	2.25	1.24
**Variance**	0.38	1.07	0.56	2.50	5.08	1.53
**CV**	0.08	0.20	0.11	0.52	0.93	0.13

**Table 3:** Descriptive statistics of 18s ribosomal unit reference gene based on its crossing point (CP) values. Abbreviations: N: number of samples; GM: the geometric mean of CP; AM: the arithmetic mean of CP; Min CP and Max CP: the extreme values of CP; SD: the standard deviation of the CP; CV: the coefficient of variance expressed as a percentage on the CP level.

	Spleen MRL	Kidney MRL	UUO	IRI
**N**	41	55	25	25
**GM**	9.38	7.86	9.22	9.02
**AM**	9.40	7.91	9.23	9.03
**Minimum**	8.10	6.44	8.36	7.95
**Maximum**	10.90	9.82	10.41	9.89
**SD**	0.70	0.88	0.52	0.56
**Variance**	0.48	0.77	0.27	0.31
**CV**	0.05	0.06	0.03	0.03

**Table 4:** Gene-specific primers with example efficiencies (shown for the pooled various organs cDNA samples) and accessions numbers.

	Mouse right primer sequence	Mouse left primer sequence	Accession Nr.	Efficiency	Error value
**Dectin-1**	GTGCAGTAAGCTTTCCTGGG	TCCCGCAATCAGAGTGAAG	NM_020008	1.99	0.0007
**MR1**	GTGGATTGTCTTGTGGAGCA	TTGTGGTGAGCTGAAAGGTG	NM_008625	2.16	0.0140
**MR2**	CTCCAGACAGCCCTGCAT	GTCTTGCTTCTCGGGGGACT	NM_008626	1.81	0.0156
**DC-SIGN**	CTGCACAGTCTTCCTCTCCC	TGGTACTGGGTAGATGGTTCA	NM_133238	2.00	0.0188
**Syk**	TCTGCACCCCTTCAGAGTTC	TCCTTTCAACGTTCCATGCT	NM_011518	1.79	0.0510
**Card-9**	ATGAGGCTGTGCCTGAGC	GCTGCAAGGACGAGAACTATG	NM_001037747	2.08	0.0174
**Bcl-10**	TGCACGTAGATGATCAAAATGTC	ACGGAGGAGGATTTGACTGA	NM_009740	2.13	0.0740
**Malt-1**	CAAAAGGATGTCCAGTTGCC	CACACTGAGGTTCTTCCGCT	NM_172833	1.82	0.0065
**Src**	TACCACTCCTCAGCCTGGAT	ACACGAGGAAGGTGGATGTC	NM_009271	2.19	0.0374
**Dec-205**	TTCAGACCAATCCACAACCA	AGCTCACCTACCCAGCTTCA	NM_013825	2.03	0.1900
**Galectin-1**	GCGAGGATTGAAGTGTAGGC	AATGTCTCAAAGTTCGGGGA	NM_008495	1.96	0.0085
**Tim-3**	AGCCCATGTGGAAATTTTTG	CTCCAAGAACCCTAACCACG	NM_134250	2.00	0.0145
**Trem-1**	CACTGTCAAAGTCTGGCCCT	ACTGCTGTGCGTGTTCTTTG	NM_021406	2.00	0.0740
**DAP-12**	TTGCCTCTGTGTGTTGAGGT	CGGAAACAACACATTGCTGA	NM_011662	1.90	0.0379

	**Human right primer sequence**	**Human left primer sequence**	**Accession Nr**	**Efficiency**	**Error value**

**Dectin-1**	GGAGATGGGTTTTCTTGGGT	GACTGAGGTACCATGGCTCTG	NM_022570	2.06	0.0541
**MR1**	CCCATCGGAATTTCTGTGAT	GGGTGCTGTTCTCCTACTGG	NM_002438	2.06	0.0587
**MR2**	CAGTCCATGGCTGAAGATGA	GCTGCGTCCTGCTCCTC	NM_006039	2.22	0.1030
**DC-SIGN**	TTGTTGGGCTCTCCTCTGTT	AAGTAACCGCTTCACCTGGA	NM_021155	2.00	0.0122
**Syk**	AAAGAAGGGCAGGTGGTTG	GAGAGCGAGGAGGAGCG	NM_003177	2.07	0.0197
**Card-9**	CTGTGCGTGCAGCTCCT	TCCAAGATGTACAAGGACCG	NM_052813	2.10	0.2160
**Bcl-10**	TTGCACGTAGATGATCAAAATGT	TCCCTCACCGAGGAGGAC	NM_003921	2.00	0.0178
**Malt-1**	GCCAAGACTGCCTTTGACTC	TTTCCTGCAGGCTATGGAAC	NM_006785	2.17	0.0364
**Src**	TAGTTGCTGGGGATGTAGCC	CTGTCCTTCAAGAAAGGCGA	NM_005417	2.12	0.0134
**Dec-205**	CCAGCCAAAAACTTCTCATTT	TGGCTTCATGGGTCATGTTA	NM_002349	2.01	0.2010
**Galectin-1**	AGGTTGTTGCTGTCTTTGCC	CAAACCTGGAGAGTGCCTTC	NM_002305	1.87	0.0810
**Tim-3**	GCGAATTCCCTCTGCTACTG	CTTCGGCGCTTTAATTTTCA	NM_032782	2.02	0.0914
**Trem-1**	TACTCAGGAATCCACCAGCC	CCGATGTCTCCACTCCTGAC	NM_018643	1.92	0.0731
**DAP-12**	GTCATGATTCGGGCTCATTT	GAGACCGAGTCGCCTTATCA	NM_003332	2.08	0.0191
