# Lech, M., *et al.*, Quantitative Expression of C-Type Lectin Receptors in Humans and Mice. *Int. J. Mol. Sci.* 2012, *13*, 10113–10131.

**DOI:** 10.3390/ijms131217294

**Published:** 2012-12-18

**Authors:** Maciej Lech, Heni Eka Susanti, Christoph Römmele, Regina Gröbmayr, Roman Günthner, Hans-Joachim Anders

**Affiliations:** Medical Clinic and Policlinic IV, Nephrological Center, University of Munich, 80336 Munich, Germany; E-Mails: heni_eka.Susanti@med.uni-muenchen.de (H.E.S); christoph.roemmele@med.uni-muenchen.de (C.R.); regina.gröbmayr@med.uni-muenchen.de (R.G.); roman.günthner@med.uni-muenchen.de (R.G.); hjanders@med.uni-muenchen.de (H.-J.A.)

The authors wish to add this correction on their paper published in *IJMS*[1]. Galectin-1 was misclassified as a C-type lectin. Galectin-1 belongs to the family of the S-type lectins, *i.e.*, the galectins. These errors have been amended in an amended version of the manuscript, which is available from the International Journal of Molecular Sciences website. The authors and publisher apologize for the inconvenience.

The corrected version can be accessed at: http://www.mdpi.com/1422-0067/13/12/17294/s1.
