# Supplementary material for: Quantitative Expression of C-Type Lectin Receptors in Humans and Mice
Source: Int J Mol Sci. 2012 Aug 14;13(8):10113–31. doi: 10.3390/ijms130810113 (PMC3431848; doi:10.3390/ijms130810113)
Supplement: Supplementary file 1 [file ijms-13-10113-s001.pdf]

Supplementary table 1

	mouse right primer sequence	mouse left primer sequence
dectin-1	GTGCAGTAAGCTTTCCTGGG	TCCCGCAATCAGAGTGAAG
MR1	GTGGATTGTCTTGTGGAGCA	TTGTGGTGAGCTGAAAGGTG
MR2	CTCCAGACAGCCCTGCAT	GTCTTGCTTCTCGGGGGACT
DC-SIGN	CTGCACAGTCTTCCTCTCCC	TGGTACTGGGTAGATGGTTCA
Syk	TCTGCACCCCTTCAGAGTTC	TCCTTTCAACGTTCCATGCT
Card9	ATGAGGCTGTGCCTGAGC	GCTGCAAGGACGAGAACTATG
Bcl10	TGCACGTAGATGATCAAAATGTC	ACGGAGGAGGATTTGACTGA
Malt1	CAAAAGGATGTCCAGTTGCC	CACACTGAGGTTCTTCCGCT
Src	TACCACTCCTCAGCCTGGAT	ACACGAGGAAGGTGGATGTC
DEC205	TTCAGACCAATCCACAACCA	AGCTCACCTACCCAGCTTCA
Galectin-1	GCGAGGATTGAAGTGTAGGC	AATGTCTCAAAGTTCGGGGA
TIM3	AGCCCATGTGGAAATTTTTG	CTCCAAGAACCCTAACCACG
TREM1	CACTGTCAAAGTCTGGCCCT	ACTGCTGTGCGTGTTCTTTG
DAP12	TTGCCTCTGTGTGTTGAGGT	CGGAAACAACACATTGCTGA

	human right primer sequence	human left primer sequence
dectin-1	GGAGATGGGTTTTCTTGGGT	GACTGAGGTACCATGGCTCTG
MR1	CCCATCGGAATTTCTGTGAT	GGGTGCTGTTCTCCTACTGG
MR2	CAGTCCATGGCTGAAGATGA	GCTGCGTCCTGCTCCTC
DC-SIGN	TTGTTGGGCTCTCCTCTGTT	AAGTAACCGCTTCACCTGGA
Syk	AAAGAAGGGCAGGTGGTTG	GAGAGCGAGGAGGAGCG
Card9	CTGTGCGTGCAGCTCCT	TCCAAGATGTACAAGGACCG
Bcl10	TTGCACGTAGATGATCAAAATGT	TCCCTCACCGAGGAGGAC
Malt1	GCCAAGACTGCCTTTGACTC	TTTCCTGCAGGCTATGGAAC
Src	TAGTTGCTGGGGATGTAGCC	CTGTCCTTCAAGAAAGGCGA
DEC205	CCAGCCAAAAACTTCTCATTT	TGGCTTCATGGGTCATGTTA
Galectin-1	AGGTTGTTGCTGTCTTTGCC	CAAACCTGGAGAGTGCCTTC
TIM3	GCGAATTCCCTCTGCTACTG	CTTCGGCGCTTTAATTTTCA
TREM1	TACTCAGGAATCCACCAGCC	CCGATGTCTCCACTCCTGAC
DAP12	GTCATGATTCGGGCTCATTT	GAGACCGAGTCGCCTTATCA
